# The overlap between vascular disease and Alzheimer’s disease - lessons from pathology

**DOI:** 10.1186/s12916-014-0206-2

**Published:** 2014-11-11

**Authors:** Johannes Attems, Kurt A Jellinger

**Affiliations:** Institute of Neuroscience, Newcastle University, Campus for Ageing and Vitality, Newcastle upon Tyne, NE4 5PL UK; Institute of Clinical Neurobiology, Medical University Vienna, Kenyongasse 18, Vienna, 1070 Austria

**Keywords:** Alzheimer’s disease, Cerebrovascular lesions, Cerebral amyloid angiopathy, Cognitive impairment, Lacunes, Microinfarcts, Small vessel disease, White matter lesions

## Abstract

Recent epidemiological and clinico-pathological data indicate considerable overlap between cerebrovascular disease (CVD) and Alzheimer’s disease (AD) and suggest additive or synergistic effects of both pathologies on cognitive decline. The most frequent vascular pathologies in the aging brain and in AD are cerebral amyloid angiopathy and small vessel disease. Up to 84% of aged subjects show morphological substrates of CVD in addition to AD pathology. AD brains with minor CVD, similar to pure vascular dementia, show subcortical vascular lesions in about two-thirds, while in mixed type dementia (AD plus vascular dementia), multiple larger infarcts are more frequent. Small infarcts in patients with full-blown AD have no impact on cognitive decline but are overwhelmed by the severity of Alzheimer pathology, while in early stages of AD, cerebrovascular lesions may influence and promote cognitive impairment, lowering the threshold for clinically overt dementia. Further studies are warranted to elucidate the many hitherto unanswered questions regarding the overlap between CVD and AD as well as the impact of both CVD and AD pathologies on the development and progression of dementia.

**Electronic supplementary material:**

The online version of this article (doi:10.1186/s12916-014-0206-2) contains supplementary material, which is available to authorized users.

## Introduction

The interaction between cerebrovascular disease (CVD) and Alzheimer’s disease (AD) is a topic of considerable current interest. With age there is an increasing prevalence of coincident AD and CVD that is well recognized. Since 50% to 84% of the brains of persons who die aged 80 to 90+ show appreciable cerebrovascular lesions (CVL) [1], a specific problem is their impact in relation to AD pathology [2]-[8]. CVD frequently occurs in brains of both non-demented elderly and AD patients. The burden of vascular and AD-type pathologies are leading and independent causes of dementia in the elderly [4],[9]-[15], suggesting additive or synergistic effects of both types of lesions on cognitive impairment [2],[3],[5],[9],[16]-[29].

Epidemiological studies have shown that AD and CVD share common risk factors such as hypertension during midlife, diabetes mellitus, smoking, apolipoprotein E (ApoE) ε4 isoforms, hypercholesterolemia, homocysteinemia, and, in particular, age [16],[30]-[34]. Cardiovascular risk factors, e.g., atrial fibrillation and congestive heart failure, have also been linked to the pathogenesis and progression of AD and are among the most important modifiable risk factors for AD [35]-[42]. In the Medical Research Council Cognitive Function and Ageing Study, vascular risk factors were not associated with an increased burden of AD pathology at death in old age, whereas cerebral small vessel disease (SVD) and cardiovascular disease were interrelated [43]. According to other studies, non-stroke cardiovascular disease increases the risk of late-life dementia but it is only a risk factor for AD in carriers of the ApoEε4 allele, while the association between cardiovascular disease and dementia is not explained by genetic or early life environmental factors common to both disorders [44]. AD patients with concomitant CVD were reported to be older and more severely demented, but have less severe AD pathology than patients without CVD [23],[45].

## Review

### Coincidence between cerebrovascular disease and Alzheimer’s disease

There is a large body of literature regarding coincidence or overlap of CVD and AD and its correlation with dementia [1],[4],[5],[9],[10],[46]-[48]. Of note, this association was recently found to be stronger in cases with lower neurofibrillary tangle pathology (i.e., lower neuritic Braak stages) [5], similar to earlier studies on respective associations with subcortical vascular pathology [6] and general CVD [1]. However, others found an inverse relation between neuritic Braak stage and cerebrovascular pathology in AD [49]. A recent study assessed CVD in 5,715 autopsy cases of the National Alzheimer’s Coordinating Center (NACC) database, and confirmed previous data on the prevalence of CVD in AD and the additive or interactive deleterious effect of both AD and vascular pathologies on cognition [6],[9],[47],[50],[51]. However, the role of combined cerebrovascular pathology and AD in dementia is still under discussion and data obtained from epidemiological and clinico-pathological studies regarding their relation are controversial [13],[17],[22],[23],[52]-[55].

AD has been reported to present frequently together with SVD, microvascular injury, and microscopic CVLs [8],[16],[47],[56]-[60]. SVD-induced ApoE leakage was associated with AD and accumulation of β-amyloid (Aβ) in perivascular astrocytes [61] and transient induction of Aβ deposition [62]. CVD has been shown to induce Aβ deposition, which may by itself cause CVD, in particular micro-vascular degeneration [63]. In addition, aging, *per se*, has an effect on cerebral arteries in relation to AD since such age related changes may impair the drainage of soluble Aβ out of the brain, which in turn leads to Aβ accumulation in vessel walls and brain parenchyma associated with perturbation of cerebral perfusion and loss of homeostasis of the neuronal environment due to energy failure [64],[65]. It was also suggested that more Aβ accumulates with age in brains of vascular dementia (VaD) subjects compared to elderly without CVD [66].

Activity of smooth muscle actin (SMA) was reduced in the brains of patients with late stage AD, while increased arteriolar SMA expression together with frequent Aβ plaques observed in the brains of non-demented subjects suggests that increased SMA expression might represent a physiological response to neurodegeneration that could prevent or delay the onset of clinical dementia in subjects with cerebral AD neuropathology [67]. Vascular disease is thought by many authors to play a major role in the pathogenesis of AD and some even consider AD as being rather a primarily vascular than a neurodegenerative disorder [22],[68]-[74]. Cerebral hypoperfusion-inducing cortical microinfarcts may further aggravate cognitive decline in AD [75]. However, AD pathology alone more frequently accounts for dementia than both macroscopic and microscopic infarcts [15] and, in late stages of AD, concomitant SVLs do not significantly influence the overall state and progression of cognitive decline [45],[54],[76], the severity and extent of AD pathology overwhelming the rather modest influence of CVD on cognitive impairment [8],[77],[78]. These data add further evidence for AD pathology (mainly neurofibrillary tangles and neuritic plaques) being the main morphological substrate of clinical dementia [51],[79],[80]. On the other hand, CVD has been associated with worse cognitive performance in AD and neuropathological studies report that CVD lowers the threshold for dementia in subjects with a pathological diagnosis of AD [5],[6],[8],[9],[13],[17],[23],[51],[53],[81]-[83]. CVD has been suggested to contribute to AD neuropathological changes including selective brain atrophy and accumulation of abnormal proteins such as Aβ [24],[35],[84],[85]. Moreover, AD pathology and subcortical vascular disease may independently affect cortical atrophy [86].

### Vascular pathology in aging and Alzheimer’s disease

The types of vascular pathology in the aged human brain include:

Cerebral amyloid angiopathy (CAA);

Cerebral atherosclerosis, SVD (in most cases caused by hypertension, i.e., hypertensive vasculopathy), or microvascular degeneration (tortuosity, fibro- and lipohyalinosis,);

Blood-brain barrier (BBB) dysfunction causing white matter lesions (WMLs), microinfarctions, lacunes or lacunar infarcts, and microbleeds [17],[87].

### All of these pathologies may disrupt the integrity of cerebral vessels and alter brain perfusion leading to neuronal injury and cognitive impairment

CAA results from focal to widespread deposition of Aβ within leptomeningeal and intracortical arteries, arterioles, capillaries, and, rarely, veins causing fibrinoid necrosis, intimal thickening, and microaneurysms. In addition, pericapillary Aβ refers to Aβ depositions in the glia limitans and adjacent neuropil, whereas in capillary CAA Aβ depositions are present in the capillary wall [88]. Sporadic CAA is present in 82% to 98% of AD patients, often associated with ApoE2 and ApoE4 alleles [80], but is also frequently observed in brains of elderly non-demented individuals with an age-related prevalence between 10% and almost 100% [17],[89]. The occipital lobe has been reported to be the site most frequently and severely affected by CAA, followed by either frontal, temporal, or parietal lobes [89],[90]. CAA may cause lobar intracerebral hemorrhages (ICH) and microbleeds [91]; it is indeed considered a risk factor for non-traumatic ICHs in the elderly and is present in up to 20% of all cases with ICH [92]. However, in a large autopsy cohort, the prevalence of ICH was similar in cases with and without CAA (around 5%) [93],[94]. Of note, the majority of cases with CAA-related ICH had hypertension, suggesting that hypertension is an important additional causal factor in CAA-related ICHs [95],[96]. The progression of WMLs in subjects with CAA has been associated with incident lobar ICHs [97]. CAA has been suggested to cause cortical microinfarcts [98],[99], while others did not confirm such an association [100]. Moderate to severe CAA is considered to be an independent risk factor for cognitive impairment [101].

The clinical diagnosis of CAA is based on the assessment of associated CVLs by magnetic resonance imaging (MRI)/cranial computerized tomography (CCT) and clinical data. Correlations of these criteria with post-mortem neuropathological findings indicate that the diagnosis of probable CAA-related hemorrhage can be made *intra vitam* with high accuracy [102]-[105]. In addition to the presence of superficial siderosis, cerebral microbleeds, cortical microinfarcts, and hypointensities in MRI images [106]-[109], the use of Pittsburgh Compound-B (PiB)-positron emission tomography (PET) is useful in detecting CAA *intra vitam*[110],[111], and a significant decrease of both Aβ-40 and Aβ-42 in cerebrospinal fluid (CSF) may prove useful in the diagnosis of CAA [112],[113], while in AD, Aβ-42 but not Aβ-40 are significantly decreased [114].

SVD affects small arteries and arterioles and refers to pathological changes similar to atherosclerosis that are termed small vessel arteriosclerosis/atherosclerosis, lipo- or fibrohyalinosis, or hypertensive arteriopathy [115]. They are common in basal ganglia and in the white matter, while small brainstem arteries usually develop arteriosclerosis only in end stages of SVD and cortical vessels usually do not show signs of SVD [116]. In AD neither Aβ load nor metabolic deficit are dependent on the age of disease onset, but patients with late-onset AD show a significantly higher amount of SVD that influences the association between metabolic deficit and clinical symptoms [117]. SVD is a frequent cause of white matter lesions (WMLs; leukoaraiosis) that are increasingly detected by neuroimaging [118]-[121]. Enlarged perivascular spaces in the centrum semiovale are MRI markers indicative of CAA (in the overlying cortex), while those in basal ganglia are usually associated with hypertensive arteriopathy [103],[104]. Deep cerebral microbleeds (CMB) are mainly linked to subcortical SVD, while both subcortical SVD and CAA interact to increase the risk of lobar CMBs [122],[123]. The associated morphological findings include demyelination, axon loss, lacunar infarcts, or enlarged perivascular spaces, most frequently in the frontal, parietal, and occipital white matter [124]. Frontal lobe WMLs have been shown to be associated with neurofibrillary pathology, particularly in the oldest old, while there was no relationship with neocortical Aβ load [125]. Routine histological assessment may underrate mild to moderate subcortical vascular lesions, but MRI imaging of fixed *post-mortem* brains reliably reflects subcortical vascular pathology of the white matter [126],[127].

BBB dysfunctions related to SVD leading to a leakage of plasma proteins into enlarged perivascular spaces [61],[128] have been described in WMLs and lacunar stroke [129],[130]. These observations point towards SVD-related alterations of the pre-capillary BBB segment which are involved in the pathogenesis of WMLs/lacunar infarcts and associated with vascular lesions in addition to AD-related changes [61],[116]. Thus, chronic plasma protein leakage into the brain and retention of extracellular fluid due to altered perivascular clearance may contribute to the development of WMLs and/or lacunar infarcts [2],[3],[87]. Damage to the vasculature may, in turn, impair the BBB integrity as one mechanism by which WMLs may evolve [124]. Mechanisms leading to BBB leakage in aging brains are complex, including oxidative damage and the activation of proteases, matrix metalloproteinases, and cyclooxygenases [131]. Evidence of early increase of BBB changes and their progression with severity of AD-type pathology suggest that BBB dysfunction contributes to damage in the aging brain [132].

Atherosclerosis is a very common vessel disorder in elderly individuals, frequently affecting large- to medium-sized arteries of the entire cardiovascular system (large-vessel disease; LVD). With respect to the cerebrum, it mainly affects the circle of Willis and the carotid arteries, in particular at the level of the carotic bifurcation. It causes narrowing of the arteries’ lumina, thereby reducing the blood blow for the supported region, while rupture of atherosclerotic plaques often leads to thrombosis that results in either occlusion of the vessel or thromboembolisms. Depending on the size of the embolus, it may cause lesions that range from “silent” infarcts or microinfarcts to large cerebral infarcts with overt clinical symptoms. “Silent” lacunar infarcts are frequently detected by MRI or CCT and are not accompanied by any overt clinical symptoms, but double the risk of subsequent stroke and dementia [133]. They have been shown to be associated with atrophy in multiple subcortical structures, ventricular enlargement, and widespread cortical thinning, supporting the assumption of a vascular contribution to neurodegeneration and cognitive impairment [134]. As opposed to large and lacunar infarcts, cortical microinfarcts (CMI) are usually not visible at gross neuropathological examination. Due to the location of the underlying vessel disorder, multiple cortical CMIs are often associated with CAA, whereas subcortical microinfarcts are mainly linked to SVD or atherosclerosis-related embolism [135]. A systemic review of CMIs reported frequencies of 43% in patients with AD and 24% in non-demented older adults [136], while a 7-Tesla MRI study revealed CMI occurrence in 55% of early AD and 45% of non-demented age-matched controls [137].

Widespread CAA and SVD have been suggested to contribute to neurodegeneration in AD [116]. Moreover, atherosclerosis in the circle of Willis has been specifically linked to AD [138]-[140], and the presence of large-vessel CVD was strongly associated with an increased frequency of neuritic plaques, suggesting a common etiology or a reciprocal regulation for atherosclerosis and AD [138],[141]. Others, however, saw no direct association between large-vessel cerebral atherosclerosis and AD pathology [142], suggesting that atherosclerosis of the intracranial vessels is an independent and important risk factor for dementia due to potentially reversible pathways unrelated to AD pathology and stroke [143]. The pathophysiology of VaD has been critically reviewed recently [48],[144]-[146].

### Topographical distribution of cerebrovascular lesions

In AD brains with minor CVD the majority of CVLs are lacunar infarcts in basal ganglia and white matter, and multiple micro-infarcts. This pattern of topographical distribution of CVLs is very similar to the one seen in “pure” vascular dementia (VaD without AD pathology beyond age-related lesions), where around 68% are lacunar infarcts in subcortical brain areas or strategic infarcts involving the thalamus or hippocampus, whereas only 32.5% were multiple large cortico-subcortical infarcts (Table 1). By contrast, mixed dementia (AD + severe CVD), according to our experience, is more frequently characterized by large or lobar infarcts, and multiple cortico-subcortical lesions (56.6%) than small subcortical lacunar infarcts, micro-infarcts, or strategic infarcts (43.4%, Table 2), suggesting different pathogenic mechanisms between these types of disorders [2],[3]. In both pure VaD and AD + minor CVD, microangiopathy (SVD) appears more important than in mixed dementia. The type and average prevalence of CVLs in AD, VaD, mixed dementia, and aged controls is shown in Table 3[147]. The combination of two or more pathological processes may influence the severity of cognitive deficits, unmasking preclinical dementia due to mild AD lesions, while small CVLs alone, seen in 10% to 50% of aged cognitively unimpaired controls, are not likely to account for a single cause of dementia.

**Table 1:** **Types and location of cerebrovascular lesions in vascular dementia (total 188)**

**Multiple infarcts (61 = 32.5%)**	
MCA bilateral	4
MCA left/right	9
MCA bilat. + PCAS/PCAD	2/1
MCA bilat. + PCA bilat.	2
MCAS + PCAS	4
MCAD + PCAD	4
PCA bilateral	3
PCA left/right	5/7
ACAS + MCAS	2
ACAD	1
Multiple cortico-subcortico bilateral	12
Multiple cortico-subcortico left hem.	2
**SAE (subcortical) (108 = 57.4%)**	
Basal ganglia	21
Basal ganglia + white matter	31
Basal ganglia + thalamus (+white matter)	33
Basal ganglia brainstem (+thalamus)	23
**SID/strategic infarcts (19 = 10.1%)**	
Thalamus bilateral	9
Thalamus left	2
Thalamus + hippocampus	8

**Table 2:** **Types and location of cerebrovascular lesions in mixed dementia (n = 83)**

**1) AD + Multiple infarcts (47 = 56.6%)**	
MCA bilateral	7
MCA left	6
MCA right (+ lacunes basal ganglia)	3/1
MCA + ACA bilat.	1
MCA + PCA left	2
MCA + PCA right	1
MCA + PCA left/right	3/3
MCA bilat. +PCAD	1
PCA bilateral	2
Multiple cort. and subcort. bilateral	13
Multiple left hemisphere	4
**2) AD + SAE (subcortical) (33 = 39.8%)**	
Lacunes basal ganglia	15
Lacunes basal ganglia + white matter	8
Lacunes basal ganglia + thalamus	10
**3) AD + SID/strategic infarcts (3 = 3.6%)**	
Thalamus bilateral	2
Thalamus + hippocampus	1

**Table 3:** **Common lesions in AD, VaD, MIX, and aged controls (from**[130]**)**

Pathological feature	AD [%]	VaD [%]	MIX [%]	Aged controls [%]
Cerebral amyloid angiopathy	98	30	~90	23-45
Small vessel disease/MVD	~50	>50	>50	~20
Total infarctions	10-20	100	30-40	>10
Microinfarcts/lacunes	30-46	70	60-70	17-21
Intracerebral hemorrhage	10-15	15	10	1-2
White matter pathology	40	80	70-80	<20
Loss of cholinergic markers	75	40	~70	
CVD/atherosclerosis	45-60	60	~60	30-53

Abbreviations: AD, Alzheimer’s disease; CVD, Cerebrovascular disease; MIX, mixed type dementia (AD plus vascular dementia); MVD, Microvascular disease; VaD, Vascular dementia.

### Cerebrovascular and Alzheimer’s disease pathology in demented and non-demented elderly

In a series of 300 autopsy cases of AD, Kalaria and Ballard [148] reported 98% CAA, 100% microvascular degeneration, 31% infarcts of all sizes, and 7% intracerebral hemorrhage, while Olichney [149], in a cohort of 248 autopsy cases of AD, revealed a total of 48% CVLs, with 31% microinfarcts, 12.5% large infarcts, and 13.5% hemorrhages. Comparing 173 autopsy-proven AD cases and 130 age-matched controls, CVL were significantly less frequent in controls (42.4%) as compared to AD (56.4%, *P* <0.05), and CAA was seen in 97.2% of AD cases, out of which 26% showed severe degrees [150]. In a population-based study of 419 demented persons, with neuropathological data available in 89 (21%), the neuropathological diagnoses were AD (51%), VaD (13%), combined AD + VaD (12%), and others (24%). Criteria for pure VaD using imaging results (Mayo Clinic criteria) showed 75% sensitivity and 81% specificity [151]. In a UK population-based autopsy study on elderly subjects (n =209, 48% demented), neuropathological evidence of CVD was found in 78% and of AD in 70%. The proportion of multiple CVL was higher in the demented group, while only 21% of clinically-demented patients showed “pure” AD pathology at *post-mortem*, indicating that most patients had mixed disease [152]. In a retrospective series of 730 autopsy cases of AD and 535 age-matched controls, using a four-grade scale for the severity of CVLs, the total prevalence of CVD in AD was significantly higher than in controls (31.6% vs. 23.4%) [153]. In a population based longitudinal study of over-80-year-old brain donors from Cambridge, UK, 53% of subjects presented with clinical dementia. In those cases, neuropathological findings were consistent with AD in 67% and with pure VaD in 4%, while 22% showed mixed pathologies and 1% dementia with Lewy bodies. AD and CVD frequently co-existed in the very old [154]. Among 190 older autopsy cases, 68% had CVLs, vascular score was associated with dementia (OR, 1.6), AD (OR, 1.5), and VaD (OR, 2.0). Leukoencephalopathy, large infarcts, and higher vascular burden were associated with clinical dementia [18]. Analysis of 4,629 cases of the NACC database with autopsy-confirmed neurodegenerative AD classified 79.7% as having CVD [37].

In a recent study from the Oxford Project to Investigate Memory and Ageing, assessment of the severity of SVD in 161 cases of autopsy-confirmed AD gave no relationship between the SVD score and cognitive scores acquired in the last two years of life nor to blood pressure at entry; further, SVD scores were significantly lower when compared with a cohort of cases with only CVD [8]. Assessment of 175 autopsy cases in the Baltimore Longitudinal Study of Aging cohort found no relationship between the degree of atherosclerosis in the aorta, heart, and intracranial vessels and the degree of AD pathology, while the presence of intracranial atherosclerosis significantly increased the odds of dementia, independent of cerebral infarction [143].

A recent study from the NACC selected 835 subjects that represent the AD continuum. While the cause of mild to moderate dementia remained uncertain in 14% of the patients, plaques and tangles independently predicted cognitive dysfunction, as did severe SVD, CAA, and hippocampal sclerosis. Thus, concomitant CVD strongly correlated with cognitive impairment in this sample selected to represent the AD pathology continuum, confirming the uncertainty of AD clinico-pathological correlations based only on neurofibrillary tangles and Aβ-plaques [155]. Assessment of 856 participants of two longitudinal clinico-pathological studies (Rush Memory and Aging Project and Religious Orders Study, autopsy rate 80%, mean age at death 88.2 ± 6.5 years) showed that global AD pathology, Aβ-plaques, neurofibrillary tangles, macroscopic infarcts, and neocortical Lewy bodies were associated with faster rates of decline and explained 22%, 6%, 34%, 2%, and 8% of the variation in decline, respectively. However, much of the variation in cognitive decline remains unexplained, suggesting that other important determinants of cognitive decline remain to be identified [156].

In a consecutive autopsy series of 494 cases (257 autopsy-proven AD, mean age 83.1 ± 8.4 years and 237 age-matched non-demented controls), 42.7% of the AD brains, all showing advanced AD pathology, were free of essential vascular pathology except for minor to moderate CAA (50%) and without CVLs, compared to 66.8% in age-matched controls, all showing low Braak stages (*P* <0.01). Prevalence of CAA in AD was 94.1% (45% severe degrees) as compared to 33.3% in controls. The severity of CAA was significantly higher in AD brains with CVLs compared to controls with similar vascular lesions [157]. Minor and moderate vascular pathology in AD were about twice as frequent as in controls (26.2% vs. 12.2% and 20.9% vs. 11.3%; *P* <0.01). On the other hand, severe vascular pathology did not significantly differ between both groups (10.2% vs. 12.2%). Retrospective examination of the prevalence of CVD in a consecutive autopsy series of 621 autopsy-proven AD cases and 486 age-matched controls, using a four-degree scale for cerebrovascular pathology, showed a generally higher prevalence of CVLs in AD (67.8%) than in controls (29.4%); severe CVLs (old/recent infarcts and hemorrhages) were more frequent in AD (23.6%) than in controls (5.4%). Likewise, the prevalence of cortico-subcortical infarcts and subcortical vascular lesions was higher in AD (41.2%) compared to controls (11.6%) [157]. Both the incidence and severity of CVLs increased with higher neuritic Braak stages as was reported in a previous study [12]. In elderly subjects with and without dementia, the prevalence of “pure” VaD (without other cerebral pathologies) ranged from 5% to 78% and in the oldest old group from 4.5% to 46.8% [47], while the majority (24% to 93%) showed mixed pathologies [158],[159]. In the age group 70 to 90+, the prevalence of VaD increased from 13% to 44.8%, compared to AD (23.6% to 57%) and mixed dementia (2% to 86%) [47]. In contrast to AD and mixed dementia, the prevalence of pure VaD decreased after 80 years of age [145],[158].

Cerebrovascular lesions are found in the majority of late-onset AD and only in half of early-onset AD cases [160]. There are considerable differences in the pathological burden in relation to age of onset of dementia, suggesting that late onset is associated with increased vascular pathology and lower AD burden [161],[162]. However, in a 90+ study, there was extensive overlap in pathology among those with and without dementia; 22% of demented subjects did not have significant pathology to account for their cognitive impairment [163]. A specific caveat in this respect is the effect of sample selection in incident-bases dementia autopsy series [164]. Community samples tend to show greater degrees of cerebrovascular pathology as compared to hospital based samples; and the prevalence of mixed AD/CVD was higher in the community-based RUSH Memory and Aging Project (44%) than in the RUSH Religious Order Study (28%). Therefore, the type of study sample may strongly bias results and should be mentioned as a possible contribution to variability of findings.

Many studies emphasized multiple confounding pathologies in non-demented elderly subjects, in particular CVLs, e.g., small or large cerebral infarcts, lacunes, and WMLs, in up to 10% [10],[165]-[167]. Among 418 non-demented participants of the Religious Order Study (mean age 88.5 ± 5.3 years), 35% showed macroscopic brain infarcts and 14.8% arteriosclerosis, while only 37.5% were free of any CVD [168]. Various degrees of CAA have been found in up to 75% of cognitively normal seniors [167]. Among 100 non-demented elderly, mild, moderate, and severe intracranial atherosclerosis was present in 31%, 17%, and 6% of subjects, respectively. A lacunar state in basal ganglia and/or white matter was observed in 73%, hippocampal sclerosis in 3%, and mixed cerebral pathologies in 6%, whereas only 9% were free of CVLs [169]. A recent cross-sectional study in a community-based sample of 72 cognitively normal older individuals (mean age 74.9 ± 5.7 years) confirmed that a substantial number harbor neurodegeneration without Aβ burden, but association of neurodegenerative lesions with CVD can emerge through non-Aβ pathways within regions most affected by AD [170].

### Pathogenic factors

**Figure 1:**
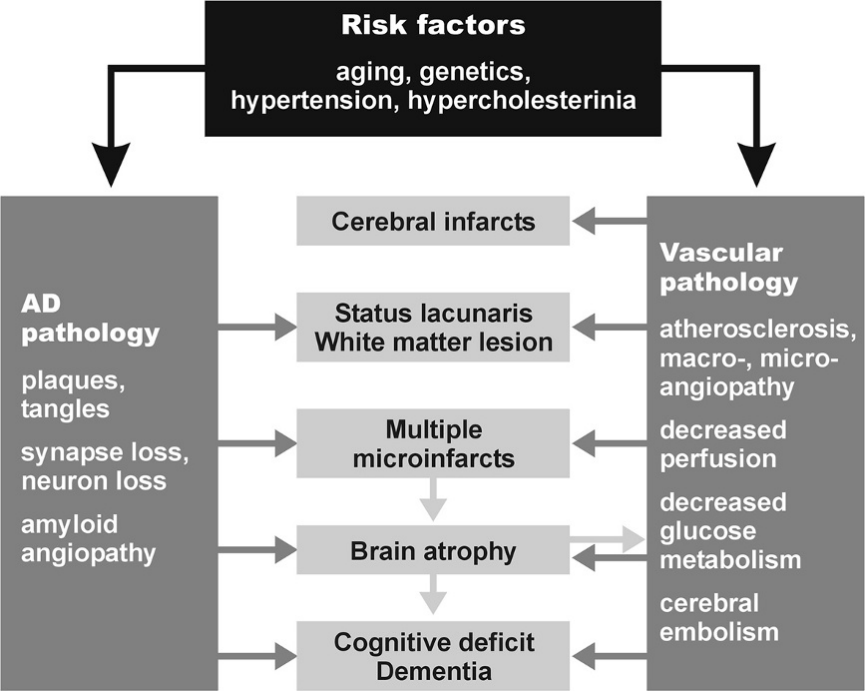
**Pathogenic factors for the development of mixed dementia.** Modified from [147].

Microvascular changes in the aged brain and in AD induce impairment of cerebral perfusion, in particular decrease of regional blood flow, reduction of glucose transport and utilization, loss of vascular innervation with special impact on the cholinergic and transmitter deficits in AD [171], impairment of neurovascular regulation, ultrastructural changes in capillaries and basement membranes due to deposition of Aβ, with breakdown of the BBB and impairment of amyloid clearance. The pathogenic chain of these and other deleterious effects, in a vicious circle, finally produces either structural cerebral disintegration (lacunes, infarcts, WMLs) with compromised neuronal metabolism, mitochondrial deficiency, oxidative stress, protein degradation, failure promoting cytoskeletal lesions with deposition of Aβ, and formation of neuritic lesions (e.g., neurofibrillary tangles). These factors induce brain atrophy with cognitive and memory impairment (Figure 1) [147], although the complex cascade of these and other noxious factors needs further elucidation.

The role of vascular pathology as a factor contributing to AD is a topic of current interest, with a wide overlap between both disorders. Both hypertension and CAA are associated with an increased prevalence of CVLs [157], and both human and experimental studies in transgenic mice overexpressing amyloid precursor protein suggest that cerebrovascular effects of Aβ render the aged brain more vulnerable to ischemic injury [172]. Both atherosclerosis and CAA cause changes in microvasculature auto-regulation and thus may lead to myelin loss, frequently seen in aged and diseased brains, suggesting shared risk factors for all pathological changes seen in AD and CVD. WMLs may be caused by both CVD (hypoperfusion) and AD (retrograde degeneration), they progress with age, and they are a considerable risk factor for cognitive impairment [120],[173],[174]. They impair frontal functions regardless of their location [175],[176] and increase the risk of dementia, particularly in patients with lacunar infarcts [177],[178], causing functional network disruption in cognitively-impaired individuals compared with age-matched healthy elderly controls [179],[180]. Although WMLs and lacunes may be independently associated with cognitive dysfunction [181],[182], WMLs in AD are significantly correlated to cortical and medial temporal lobe atrophy [181]-[183], and, thus, are assumed to contribute to cognitive decline [184]. Together with cortical microinfarcts, WMLs may contribute to the progression of cognitive impairment, but do not necessarily interact with AD pathology to increase the likelihood of dementia beyond their additive effect [20]. Further, the neuropathological evaluation of focal and white matter gliosis may have no clinical validity [185].

## Conclusions

CVD has been suggested to be an important cause of cognitive impairment in the elderly, both by itself or as a catalyst for the conversion of low-grade AD to overt dementia [186]. Hence, the combination of both AD and vascular or other pathological processes, as seen in many elderly persons, may coexist in the earlier stages of cognitive decline and may influence its progression and severity, thus representing a major diagnostic challenge not only for clinicians but also for neuropathologists. Despite multiple attempts, there is still a lack of consensus regarding the optimal means of incorporating vascular disease into clinical and neuropathological classification schemes for dementias. Therefore, an integrating rather than a strictly taxonomic approach (instead of discriminating AD, VaD, and other diseases) to elucidate specific pathophysiological mechanisms that contribute to dementia phenotypes and neuropathological causes has been proposed [37].

To improve the diagnostic specificity on the interaction between AD and CVD pathologies, a multivariable and multimodality algorithm is required. While structural MRI results have limited security and specificity, a number of *in vivo* studies using functional MRI [187] and amyloid and tau PET (e.g., PiB, florbetabin, flutemetamole, etc.) [188]-[190] will enable the identification of AD and CVD patients in clinical and research settings. However, recent evidence comparing PiB-PET with *post-mortem* or biopsy results raised doubts about this method as representative of Aβ loads in the living brain [191],[192] and PiB-positivity was observed in 55% of non-demented subjects over 80 [193]. The recent development of *in vivo* amyloid imaging enables further pathological breakdown of SVD into pure forms and mixed dementia based on the absence or presence of amyloid pathology in the brain [194]. Modern CSF biomarkers may support a direct relationship between SVD and AD pathology [195], although in the Alzheimer Disease Neuroimaging Initiative that is focused on AD, no interactions were noted between vascular risk factors and AD biomarkers [26]. Therefore, differentiation of mixed AD/CVD with CSF biomarkers may be difficult. Converging evidence from autopsy, amyloid PET, functional MRI, and CSF biomarker studies indicate that AD and CVD exert additive rather than interactive adverse effects on cognitive health, but interaction between various vascular factors and amyloidosis/tauopathy still remain unresolved. Further studies to more accurately elucidate the impact of vascular disease and AD-related brain pathology are an important challenge for neuroscience as such studies could serve as a basis for the development of efficient therapies against age associated dementias.

## Authors’ contributions

KAJ drafted the manuscript and JA critically revised the manuscript. Both authors read and approved the final manuscript.

## Authors’ original submitted files for images

Below are the links to the authors’ original submitted files for images. Authors’ original file for figure 1

## Abbreviations

Aβ: β-amyloid
AD: Alzheimer’s disease
ApoE: Apolipoprotein E
BBB: Blood-brain barrier
CAA: Cerebral amyloid angiopathy
CCT: Cranial computerized tomography
CMB: Cerebral microbleed
CMI: Cortical microinfarcts
CSF: Cerebrospinal fluid
CVD: cerebrovascular disease
CVL: Cerebrovascular lesions
ICH: Intracerebral hemorrhages
LVD: Large-vessel disease
MRI: Magnetic resonance imaging
NACC: National Alzheimer’s Coordinating Center
PiB: Pittsburgh compound-B
PET: Positron emission tomography
SMA: Smooth muscle actin
SVD: Small vessel disease
VaD: Vascular dementia
WML: White matter lesions
