# Supplementary figures and images for: The overlap between vascular disease and Alzheimer’s disease - lessons from pathology
Source: BMC Med. 2014 Nov 11;12:206. doi: 10.1186/s12916-014-0206-2 (PMC4226890; doi:10.1186/s12916-014-0206-2)

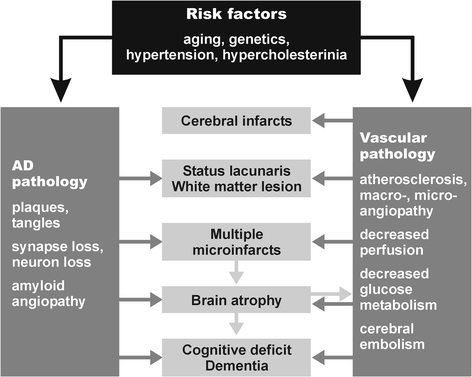

Supplement: Supplementary file 1 — Authors’ original file for figure 1 [file 12916_2014_206_MOESM1_ESM.gif]
